# Microvascular decompression for trigeminal neuralgia caused by trigeminocerebellar artery

**DOI:** 10.3171/2020.7.FOCVID2017

**Published:** 2020-10-01

**Authors:** Norio Ichimasu, Nobuyuki Nakajima, Ken Matsushima, Michihiro Kohno, Yutaka Takusagawa

**Affiliations:** 1 Department of Neurosurgery, Tokyo Medical University, Tokyo; and; 2 Department of Neurosurgery, Kosei Chuo General Hospital, Tokyo, Japan

**Keywords:** trigeminocerebellar artery, trigeminal neuralgia, surgical video, microvascular decompression, cerebellopontine angle

## Abstract

In this operative video, the authors demonstrate the case of a 53-year-old woman who presented with typical right trigeminal neuralgia by a trigeminocerebellar artery (TCA). The TCA was first defined by Marinković as a unique branch of the basilar artery supplying both the trigeminal nerve root and the cerebellar hemisphere. As a result of the close relationship between this vessel and the nerve root, the TCA might compress the nerve root, thereby causing trigeminal neuralgia. However, few cases of trigeminal neuralgia caused by TCA have been reported. This video shows the microvascular decompression for trigeminal neuralgia by the TCA.

The video can be found here: https://youtu.be/UnGsCQRK6aY

## Transcript

This video will demonstrate the microvascular decompression for trigeminal neuralgia caused by the trigeminocerebellar artery.

**0:29 Clinical History.** This 53-year-old woman was presented to our hospital suffering from facial pain on her right side. Her symptoms began 4 years previously and were characterized by an electric shooting pain in the right maxillary division of the trigeminal nerve. The pain was triggered by brushing her teeth or washing her face.

The patient was diagnosed with trigeminal neuralgia, and a treatment of oral carbamazepine was started. However, the medication did not lead to satisfactory effects, and she was admitted to our hospital for surgical intervention.

**1:08 Preoperative Radiological Images.** Preoperative axial heavy T2 imaging displayed the accumulation of flow voids next to the right trigeminal nerve. These flow voids were continuous with the intraneural space. MR angiography showed that the offending vessel arose from the upper part of the basilar artery and irrigated into the petrosal surface of the cerebellum.

Digital subtraction angiography confirmed that the offending vessel identified on MR angiography originated from the basilar artery and was perfusing the cerebellar petrosal surface.

From these anatomical features, this offending vessel was identified to be the trigeminocerebellar artery. The trigeminocerebellar artery is a branch from the upper third of the basilar artery,^1^ and it is classified into four segments: the pontine segment, the trigeminal segment, the cerebellopontine segment, and the cerebellar segment.^2^ In the trigeminal and the cerebellopontine segment near the trigeminal nerve, this artery makes several sharp turns while branching into multiple perforating arteries. Some previous studies have reported cases of the trigeminocerebellar artery penetrating into the trigeminal nerve root.^3^

**2:28 Setup and Approach.** The surgery was performed by the right retrosigmoid lateral suboccipital approach. The patient was placed in the park-bench position with the head held still by three-pin fixation using a Mayfield head holder. A V-shaped postauricular skin incision was made, and we used neuromonitoring with auditory brainstem responses.

After performing the craniotomy, the dura was opened in a Y-shaped fashion. Using the intraoperative microscope, we opened the arachnoid meticulously over the petrosal fissure and identified cranial nerves V and VIII. Around the trigeminal nerve, an offending vessel, which was the trigeminocerebellar artery, was observed, and we can see that the trigeminal nerve was compressed from the medial side to the lateral direction by this offending artery, and another direction was from the caudal side to the cranial side.

Anatomical features of the trigeminocerebellar artery, making sharp turns and having perforating branches into the brainstem, were observed. Furthermore, in this patient, the trigeminocerebellar artery ran through the trigeminal nerve.

To obtain sufficient working space, the superior cerebellar artery running along the cranial side of the trigeminal nerve was moved to the tentorial surface.

The cerebellopontine segment of the trigeminocerebellar artery was dissected and detached from the cerebellum, and a tiny perforating artery to the cerebellum was sacrificed. These procedures increased the mobility of the offending vessel as much as possible.

**4:18 Decompressive Procedures.** Care was taken not to damage the perforating arteries to the brainstem, and the trigeminocerebellar artery was moved in the caudal direction. After preparation to fix the vessel by removing the arachnoid on the petrosal bone, the offending vessel was fixed with Teflon felt and fibrin glue.

In the next step, the proximal part was fixed with Teflon felt and fibrin glue as well. The part of the trigeminocerebellar artery penetrating the trigeminal nerve was wrapped with a piece of fascia, and the operation was completed.

**5:13 Postoperative Course.** Postoperatively, the patient’s trigeminal neuralgia resolved, and she remained pain free without any medication at her 48-month follow-up.

## Author Contributions

Primary surgeon: Takusagawa. Assistant surgeon: Ichimasu, Nakajima. Editing and drafting the video and abstract: Ichimasu, Matsushima. Critically revising the work: Ichimasu, Matsushima. Reviewed submitted version of the work: Matsushima, Takusagawa. Approved the final version of the work on behalf of all authors: Ichimasu. Supervision: Nakajima, Kohno, Takusagawa.
